# Curving THz wireless data links around obstacles

**DOI:** 10.1038/s44172-024-00206-3

**Published:** 2024-03-30

**Authors:** Hichem Guerboukha, Bin Zhao, Zhaoji Fang, Edward Knightly, Daniel M. Mittleman

**Affiliations:** 1 School of Science and Engineering, University of Missouri-Kansas City, Kansas City, MO USA; 2 Department of Electrical and Computer Engineering, Rice University, Houston, TX USA; 3 School of Engineering, Brown University, Providence, RI USA

**Keywords:** Electrical and electronic engineering, Other photonics

## Abstract

A key challenge in millimeter-wave and terahertz wireless networks is blockage of the line-of-sight path between a base station and a user. User and environmental mobility can lead to blockage of highly directional beams by intervening people or objects, yielding link disruptions and poor quality of service. Here, we propose a solution to this problem which leverages the fact that, in such scenarios, users are likely to be located within the electromagnetic near field of the base station, which opens the possibility to engineer wave fronts for link maintenance. We show that curved beams, carrying data at high bit rates, can realize a link by curving around an intervening obstacle. We develop a model to analyze and experimentally evaluate the bandwidth limitations imposed by the use of self accelerating beams. We also demonstrate that such links employ the full aperture of the transmitter, even those portions which have no direct line of sight to the receiver, emphasizing that ray optics fails to capture the behavior of these near-field wave fronts. This approach, which is ideally suited for use at millimeter-wave and terahertz frequencies, opens vast new possibilities for wave front management in directional wireless networks.

## Abstract (web summary)

Hichem Guerboukha and colleagues present a reliable high-data-rate THz communications system when the line of sight between the transmitter and receiver is blocked. They design the near-field wavefront to generate a curved trajectory.

## Introduction

A common theme in the emerging vision for future wireless communication systems is the use of frequencies in the millimeter wave to terahertz band (0.1–1.0 THz)^1–3^. These high frequency bands offer the key advantage of copious bandwidth for ultrahigh data rates^4^, but also require the use of directional beams formed with high-gain antennas to overcome the free-space path loss which can easily exceed 100 dB^5^. Systems operating in the 110-170 GHz range (the waveguide D-band) are already approaching commercial viability for point-to-point directional backhaul links^6^ with ranges extending beyond 2 km^7^. Yet, realization of indoor wireless local-area networks (WLANs) remains a far greater challenge, as such systems must cope with the mobility of both network users and other individuals and objects in the broadcast sector. As a result, overcoming transient blockage events, which obstruct the line-of-sight path from a base station to a user, remains a central research challenge^8–10^. While non-line-of-sight paths can in some cases be realized by specular reflections or scattering from environmental objects^11,12^ or intelligent reflecting surfaces^13–15^, such paths are not always available with sufficient link budget to support high data rates.

In this work, we explore an alternative approach to overcoming blockage-related disruptions which relies on the fact that a typical sub-terahertz WLAN can realistically operate with all users located in the near field (the Fresnel regime) of the base station^16–22^. In contrast, legacy wireless networks that operate below 10 GHz would rarely encounter this situation (see Supplementary Note 4). For example, at a frequency of 3 GHz, the near field of a typical base station (with, for instance, a 10 cm aperture) extends only a few tens of cm from the transmitter, rendering it mostly irrelevant for wireless data transfer in a WLAN. In contrast, the same aperture operating at 300 GHz can access a near field region extending to tens of meters, easily encompassing a large room. This transition is a manifestation of the distinction between low-frequency electromagnetics and the terahertz regime, which is in many ways more akin to the realm of optics. Of course, similar ideas can also be relevant at higher frequencies, in the near infrared^23^. Yet, most free-space optical communication systems are designed for long-range links and therefore also operate in the far-field limit. It is only in the sub-terahertz range, of particular interest for 6 G networks^24^, where the value of employing near-field links becomes manifest.

By exploiting the physics of electromagnetic near fields, we demonstrate the first terahertz data links exploiting self-accelerating beams. These have the valuable property of following curved trajectories as they propagate in the near field of a transmitter^25^. Such beams have recently been studied at optical frequencies, with most efforts focused on their generation and characterization^23,26–28^ and their use in applications such as microscopy^29^, particle manipulation^30^ and laser machining^31^. However, with very few exceptions^32–34^, the consideration of near-field effects in THz networks has been limited only to the idea that a spherical wave front impinging on a large-aperture receiver can induce a phase error across the aperture due to the wave front curvature^22,35,36^. Our results demonstrate that self-accelerating near-field wave fronts can provide a valuable functionality that mitigates the challenge of blockage in a wide range of scenarios.

## Results and Discussion

### Trajectory engineering

**Fig. 1:**
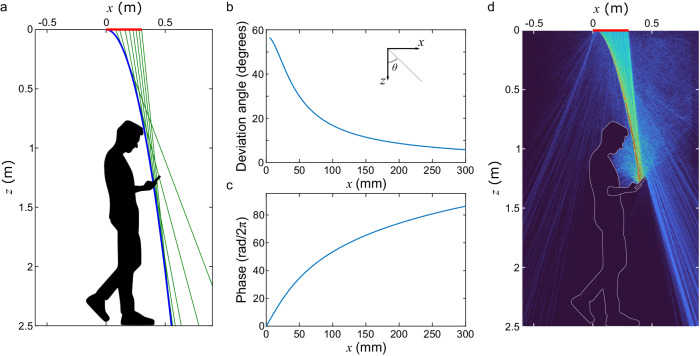
Trajectory engineering. **a** Ray optics construction of a caustic beam. The input aperture is 300 mm and shown as a red line in the ceiling. The target function $g\left(z\right)=0.3\sqrt{z}$ ($z$ in meters) is shown in blue, along with a few caustics shown in green. These caustics have an angle $\theta$ with the $z$-axis defined by $\tan \theta ={dg}\left(z\right)/{dz}$. From the generalized Snell’s law^48^, a normally incident beam deviates from its straight course according to the derivative of the phase profile, ${k}_{0}\sin \theta =d\phi (x)/{dx}$, where ${k}_{0}=2\pi /\lambda$ is the free space wavenumber. Using trigonometric considerations, one can then derive Eq. 1. **b** Required deviation angle $\theta$, and **c** corresponding phase profile $\phi (x)$ at 300 GHz. **d** Finite element method simulation depicting the near field propagation (squared electric field, ${\left|E\right|}^{2}$) when the phase is injected at the input aperture (assuming uniform amplitude). In this simulation, we assume the human silhouette to be made of water (permittivity of $\varepsilon =5.39+5.98j$^60^). For this 300-mm aperture at 300 GHz, the near-field extends to 180 m.

As a first step in engineering a system for delivering data to an obstructed user, we consider the challenge of producing a beam with the necessary curved trajectory. To illustrate our approach, consider the geometry depicted in Fig. 1a, where an access point located in the ceiling seeks to communicate with a mobile user. To do so, the access point controller designs a curved trajectory $g(z)$ that goes around the user’s head, which would otherwise significantly block the beam. Caustics – the set of lines that intersect $g\left(z\right)$ at its tangents – are defined along the curve. From these, the required angle of deviation at the input aperture can be found (Fig. 1b), and we can compute the required phase profile $\phi (x)$ of the wave emerging from each point in the emitting aperture, as:

1 $$\frac{d\phi \left(x\right)}{{dx}}=\frac{2\pi }{\lambda }\frac{{dg}(z)/{dz}}{\sqrt{1+{\left({dg}(z)/{dz}\right)}^{2}}}$$

This phase profile is shown in Fig. 1c for a frequency of 300 GHz. Figure 1d shows a finite-element method simulation in which the phase profile of Fig. 1c is injected at the input aperture. The propagation closely follows the intended trajectory, avoiding the intervening obstruction. The apparent curving behavior is a consequence of constructive interference of the caustics along the trajectory. We verified through additional simulations (see Supplementary Note 1) that the performance of this caustic beam is superior – in terms of incident power on the receiver – to both that of a steered Gaussian ( ~ 5.9 dB) and a steered focused beam ( ~ 8.6 dB). In this illustration, the function $g(z)$ is chosen merely as an example to illustrate the point, and may not be the optimal caustic trajectory, so even greater improvements may be possible.

It is worth noting that, while certain trajectories have well-established analytical solutions for the phase profile^37^, it is possible to realize any convex function. The achievable curvature is limited by the size of the aperture, such that large emitting arrays can realize more tightly curved trajectories (see Supplementary Note 2). This fact implies that there are – in principle – an infinite number of trajectories that can be engineered between two points in space, even without invoking scattering or conventional multi-path propagation. This notion completely redefines the traditional view of sparsity in line-of-sight wireless communications, which is based on a far-field conception. We therefore suggest that the use of self-accelerating waves for data transfer represents a paradigm shift for wireless networking.

**Fig. 2:**
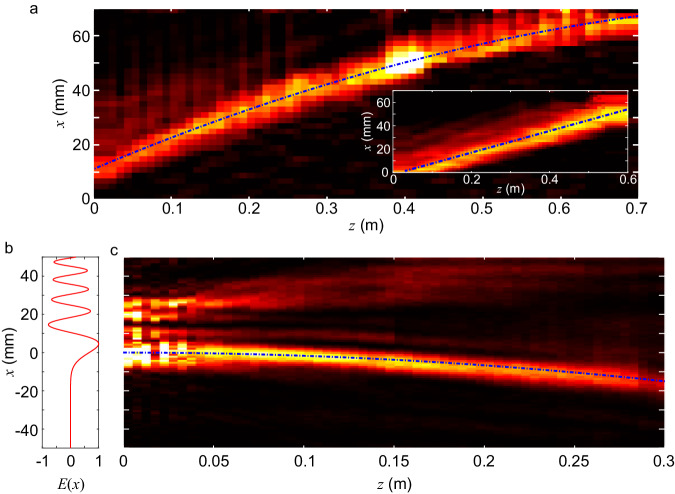
Experimental realization of THz accelerating beams. **a** Caustic beam at 200 GHz generated with a phase plate and measured with the knife edge technique (see Methods for details). The trajectory is a parabola expressed as $g\left(z\right)=-0.0001{z}^{2}+0.12z+9.8$, where $g\left(z\right)$ and $z$ are in mm. For comparison, the inset shows a steered Gaussian beam generated using a phase plate with a linear profile, which follows a straight-line trajectory (see Methods). **b** Input amplitude profile of an Airy beam generated using the beam profile $E\left(x\right)={{{{{\rm{Ai}}}}}}\left(x/{x}_{0}\right)$, where ${{{{{\rm{Ai}}}}}}(x)$ is the Airy function, and ${x}_{0}$ is a parameter that controls the curvature; here, we consider ${x}_{0}=4.5$ mm. **c** Near-field propagation (squared electric field, 200 GHz) of an Airy beam generated with a metasurface (see Methods). From the equation defining the free space propagation of Airy beams (see Supplementary Note 5), one can extract the trajectory, a parabola expressed as $g\left(z\right)=-{z}^{2}/(4{k}_{0}^{2}{x}_{0}^{3})$, shown as the dotted curve.

As shown, the caustics approach relies on an analog phase modulation at the input plane. At THz frequencies, such phase modulation can be achieved using, for example, a phase plate consisting of a transparent material of varying thickness, that locally modifies the phase of an incident beam (see Methods for details). Figure 2a displays an experimentally measured field distribution obtained from such a phase plate. The beam follows a parabolic trajectory, in contrast to the linear trajectory obtained when using a phase plate with a linearly varying thickness, which simply steers the beam due to refraction (inset of Fig. 2a). We emphasize here that Fig. 2a and its inset are both obtained with phase plates that only change the phase distribution of the incident beam, without altering its amplitude.

An alternative method of generating self-accelerating THz beams involves using both amplitude and phase modulation at the input plane^38–43^. This approach, which is amenable to implementation with, e.g., digital metasurfaces^44,45^, can be used to generate Airy beams, a diffraction-free solution to the paraxial wave equation that produces parabolic trajectories^46^. Figure 2b, c show a particular realization of an Airy beam generated via a metasurface that simultaneously modulates the amplitude and phase of an incident beam. Figure 2b shows the required amplitude and phase profile in the emitter plane, an Airy function where adjacent lobes have opposite phases and successively smaller widths.

### Near-field link budget

Exploiting near-field wave fronts such as those discussed above requires us to rethink many aspects of the engineering of wireless networks. Designing a trajectory alone is not enough; it is also necessary to perform a link budget analysis to ensure that sufficient power is delivered to the receiver. For links operating entirely in the near field of the transmitter, this procedure is very different from that used for standard far-field links.

In typical far field situations, link budgets are calculated with the Friis equation, which relates the received power ${P}_{{{{{{\rm{Rx}}}}}}}$ to the transmitted power ${P}_{{{{{{\rm{Tx}}}}}}}$:

2 $$\frac{{P}_{{{{{{\rm{Rx}}}}}}}}{{P}_{{{{{{\rm{Tx}}}}}}}}={G}_{{{{{{\rm{Tx}}}}}}}{G}_{{{{{{\rm{Rx}}}}}}}{\left(\frac{\lambda }{4\pi r}\right)}^{2}$$

where $r$ is the distance between the transmitter and receiver, and ${G}_{{{{{{\rm{Tx}}}}}}}$ and ${G}_{{{{{{\rm{Rx}}}}}}}$ are the gains of the transmitter and receiver respectively. In general, these gains are expressed as a function of the spherical coordinates $\theta$ and $\phi$, but not of the radial coordinate, since the usual $1/{r}^{2}$ dependence appears in the free-space path loss term.

**Fig. 3:**
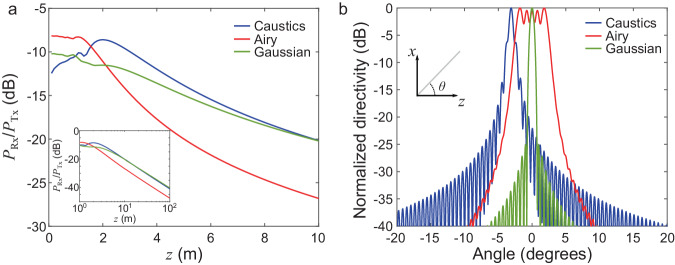
Near-field link budget. **a** as a function of the range $z$ in the near field, and (**b**) as a function of the angle in the far field. Here, the caustic corresponds to the function $g\left(z\right)=-0.0007\cdot {\left(z+20\right)}^{2}$, where $z$ is expressed in meters, the Airy beam has a curvature parameter ${x}_{0}=9.5$ mm, and the Gaussian beam has a full width at half maximum of 50 mm and a planar wave front at z = 0. This calculation is performed at 300 GHz, assuming a square input aperture of $100\times 100{{{{{\rm{m}}}}}}{{{{{{\rm{m}}}}}}}^{2}$, and in (**a**) a receiver aperture of $10\times 10{{{{{\rm{m}}}}}}{{{{{{\rm{m}}}}}}}^{2}$ centered on the maximum amplitude at each $z$ position. For simplicity in these calculations, we assume that the receiver is parallel to the input plane, and we assume that the incident field couples perfectly with the antenna. This assumption allows us to interpret the results as upper bounds on the maximal power that such a receiver can detect. More specific details about the receiver could be used to calculate the effective coupling. In the far-field, the caustic and Airy beams have calculated gains of 26.1 and 19 dB respectively, while the Gaussian beam has a gain of 27.5 dB. We note that in the far field, the Airy beam peaks in the broadside direction, while the caustic beam does not. This is a particular consequence of the fact that Airy beams are a solution to the paraxial wave equation, while caustic beams can exist in non-paraxial situations.

In the near field, however, the received ${P}_{{{{{{\rm{Rx}}}}}}}$ power does not necessarily decrease as $1/{r}^{2}$ (see Supplementary Note 3). Therefore, one cannot use this simple approach; one must instead resort to calculating the radiated field using a diffraction integral formulation of the Huygens-Fresnel principle:

3 $$\frac{{P}_{{{{{{\rm{Rx}}}}}}}}{{P}_{{{{{{\rm{Tx}}}}}}}}=\frac{\iint {{{{{{\rm{|}}}}}}{E}_{2}\left({x}_{2},{y}_{2},z\right){{{{{\rm{|}}}}}}}^{2}d{S}_{{{{{{\rm{Rx}}}}}}}}{\iint {{{{{{\rm{|}}}}}}{E}_{1}({x}_{1},{y}_{1},0){{{{{\rm{|}}}}}}}^{2}d{S}_{{{{{{\rm{Tx}}}}}}}}$$

where ${E}_{1}$ is the field at the emitter plane, ${E}_{2}$ is the field at the receiver plane, and where the integrals are performed over the receiver and transmitter apertures, ${S}_{{{{{{\rm{Rx}}}}}}}$ and ${S}_{{{{{{\rm{Tx}}}}}}}$ respectively (details about this calculation are in Supplementary Note 3). In Fig. 3, we present illustrative results for a caustic (blue) and Airy (red) beams as well as a conventional Gaussian beam (green), all transmitted (truncated) by the same aperture. Figure 3a shows the received power normalized to the transmit power (${P}_{{{{{{\rm{Rx}}}}}}}/{P}_{{{{{{\rm{Tx}}}}}}}$) as a function of the distance $z$ from the transmitting aperture for these three cases. In the near field (a few meters from the transmitter), the self-accelerating waves outperform the Gaussian beam in terms of received power. This is because the self-accelerating beams spatially focus the energy at particular locations along the bending trajectory. Interestingly, the caustic and Airy beams have distinct power behavior such that one or the other performs better at specific distances. For the specific Airy beam calculated in Fig. 3, the power dependence is relatively flat up to ~1.7 meters, followed by a drop, whereas the caustic beam focuses at ~2 meters. In comparison, the Gaussian beam has a monotonic decrease of the power as a function of $z$. These results show that the optimal beam choice depends on the position of the intended receiver.

It is also interesting to compare how these results extrapolate to the far field region. This comparison can be important for hybrid near and far-field networks where some users might be in the near-field region while others might be in the far-field, or where mobile users may move between these two regimes. The inset of Fig. 3a show this far-field behavior. In all cases, losses exceed 40 dB at z = 100 m, whereas they are only ~10 dB in the near field. This showcases another key feature of near-field networks: they require comparatively less transmit energy for operation. This is an important advantage in the THz band where efficient power generation is still an active research area^47^. As expected, the far-field results converge to the conventional $1/{r}^{2}$ dependence. There, one can use the classical Friis equation, with an angular representation of the radiation patterns and a single range-independent value of the transmitter gain. In fact, the vertical offset between these three curves at large z is a signature of their different far-field antenna gains. For the particular situations simulated here, the Gaussian and caustic beams have higher far-field gains than the Airy beam by about 7 dB, again emphasizing that different beams should be used in different situations. Meanwhile, Fig. 3b shows the far-field angular radiation patterns for the three cases shown in Fig. 3a. Here, we see that, while the Airy and the Gaussian radiation patterns are maximized at broadside (centered at 0°), the caustic beam is slightly offset. This interesting distinction arises because the caustic is generated via a nonlinear phase profile that also steers the beam in the far-field, in analogy to refraction.

### Bandwidth of Curved Beams

Transmission bandwidth is another important parameter to enable high data rate wireless communications, since link capacity grows linearly with bandwidth. The ray approach used to engineer the trajectory of caustic beams, as described earlier, is agnostic with respect to frequency. However, the corresponding phase distribution derives from the generalized Snell’s law^48^, which can be frequency-dependent. Thus, the generated beam may produce a rainbow-like pattern in free space, where for a given phase distribution $\phi \left(x\right)$, different frequencies follow different trajectories. We emphasize that this phenomenon is distinct from *angular* dispersion (also known as beam squint^49^) since it occurs in the near field, and therefore also contains a *radial* component to the spatial dispersion. This means that the produced rainbow profile can become complicated, and highly depends on the specific caustic trajectory. Furthermore, this phenomenon is different from chromatic dispersion which occurs in time and not space. Additional details on the effect of chromatic dispersion are in Supplementary Note 5.

**Fig. 4:**
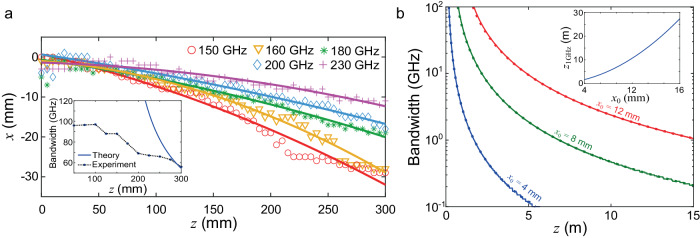
Bandwidth of an Airy beam. **a** Experimentally measured trajectories for the Airy beam shown in Fig. 2c for various frequencies in the operation range of the metasurface. The inset compares experimentally measured 3-dB bandwidth to the theory (Eq. 4, bold line) at a center frequency of 200 GHz. **b** Bandwidth as a function of $z$ when the receiver is located along the trajectory for a center frequency of 200 GHz, for various values of the curvature parameter. The calculated curves (Eq. 4, bold lines) match the results obtained via numerical simulation (dots). The inset shows the limiting range of operation to achieve a 1 GHz bandwidth as a function of the curvature ${x}_{0}$.

Figure 4a presents measured trajectories at various frequencies for the Airy beam realized in Fig. 2c. These data illustrate the rainbow effect; after propagating 300 mm, the beam deflects by ~30 mm at 150 GHz, while it deflects by only ~10 mm at 230 GHz. Consequently, receivers located at different z positions along the main lobe will collect signals with different spectral content.

For the case of an untruncated Airy beam, it is possible to derive an analytic expression for the received bandwidth, and its dependence on the curvature parameter ${x}_{0}$. For a receiver of infinitesimal size located along the main lobe trajectory generated at a center frequency ${f}_{0}$, the bandwidth evolves as:

4 $$\Delta f=\frac{z{f}_{0}}{\sqrt{{z}^{2}-2{k}_{0}^{2}{x}_{0}^{3}D}}-\frac{z{f}_{0}}{\sqrt{{z}^{2}+2{k}_{0}^{2}{x}_{0}^{3}D}}$$

where $D=1.63{x}_{0}$ is the full width at half maximum of the main lobe (see Supplementary Note 5 for details of the derivation), and where this result is valid for $z \, > \, k\sqrt{2D}{x}_{0}^{3/2}$. Figure 4b presents typical bandwidth predicted by Eq. 4 (bold lines), along with results obtained from numerical simulations (dots). For tight curvatures (small ${x}_{0}$), the bandwidth drops faster with increasing propagation, since tighter curvature implies larger spatial dispersion. The top inset shows the maximum propagation range to maintain a bandwidth larger than 1 GHz as a function of the curvature parameter; this limiting value is proportional to ${x}_{0}^{3/2}$ (see Supplementary Note 5 for details).

Of course, the frequency-dependence of the produced trajectory also depends on the generation mechanism of the accelerating beam. The result of Eq. 4 assumes that the Airy profile can be produced perfectly at all frequencies, such that the bandwidth limitation originates only from spatial dispersion associated with the curved trajectory. In any practical implementation, other factors may limit the bandwidth. For example, the C-shaped meta-atom used in the construction of the metasurface used in Fig. 2c possesses a frequency-dependent phase response, which adds additional spatial dispersion. The bottom inset of Fig. 4a compares experimentally extracted bandwidths (from results of Fig. 2c) to the analytical curve. While these show agreement in order of magnitudes, the experimental implementation shows more severe bandwidth limitations, especially near the metasurface, which are due to the non-trivial frequency-dependent phase response of the metasurface. Nonetheless, we observe bandwidths exceeding 50 GHz at all measured distances. In general, it would be possible to mitigate the effect of spatial dispersion by using an engineered frequency-encoded phase response on the metasurface^50,51^.

### Communicating Around a Wall

**Fig. 5:**
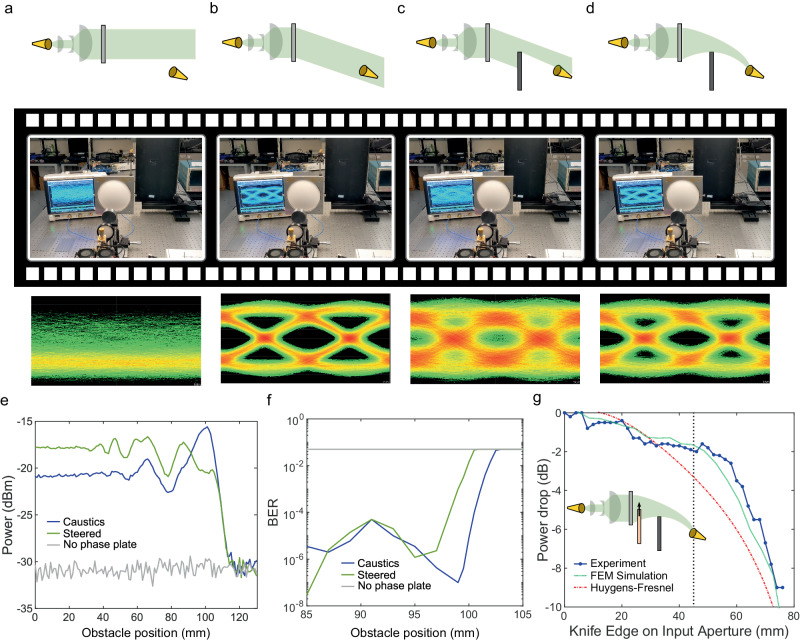
Communicating around a semi-infinite obstacle. **a**–**d** Four stages of the experiment demonstrated in Supplementary Video 1. Top shows the schematic of the corresponding frame shown in middle, for which the eye diagram is shown in bottom. **a** The receiver is located off the line of sight path, and no signal is received. **b** A phase plate with a linear phase profile is introduced in the beam which directs the beam towards receiver, producing a clear eye diagram. **c** A metallic plate is moved into the beam, partially blocking it and distorting the eye diagram. **d** The linear phase plate is replaced with a phase plate generating a caustic beam designed to curve around the metallic plate. The eye diagram improves. **e** Power measurements as a function of the obstacle position (straight edge of the metallic wall) when it is moved in the beam path, comparing the cases of using a linear phase plate (green), a caustic phase plate (blue), and when no phase plate (grey) is used (so that the beam misses the detector). **f** Corresponding measured bit error rates, on an expanded horizontal axis. **g** Power drop as a function of the position of a metallic knife edge positioned at the input plane, directly in front of the phase plate. These measurements were realized at a center frequency of 200 GHz, and using an OOK (on-off keying) modulation scheme with a bit rate of 310 Mbit/s.

Finally, we demonstrate the implementation of a communication link using a self-accelerating beam, including experimental verification of the important case of obstacle avoidance. Supplementary Video 1 shows the real-time demonstration of this experiment and is summarized in the four frames shown in Fig. 5a–d. In this experiment, the receiver is placed 1 m away from the input aperture, 10 cm away from the optical axis. In Frame #1, there is no phase plate in the beam path, so the beam continues its forward course, and we therefore measure no signal and a BER ~ 0.5 at the receiver location. In Frame #2, we place a phase plate with a linear phase profile, designed to merely steer the beam by a fixed angle towards the receiver. With this plate, we measure a strong signal of −18.4 dBm (SNR~14 dB), and a BER of $1.8\times {10}^{-7}$. When moving a metal obstacle partially into the beam path (Frame #3), the signal drops by 5 dB, enough to greatly increase the measured BER by 4 orders of magnitude. To circumvent this problem without moving the metal obstacle, we replace the steering phase plate by a phase plate designed to create a caustic beam with a trajectory that curves around the obstacle (Frame #4). This recovers 3.6 dB of power and decreases the BER by 3 orders of magnitude. In these experiments, the caustic phase plate is the same size as the phase plate with a linear profile, and both are placed at exactly the same positions in the beam path. This means that both are excited by the same incident beam i.e., they have the same amplitude profile. This ensures a fair comparison between the steered beam and the caustic beam. This sequence of results confirms that the caustic beam outperforms simple beam steering for this obstacle position.

To further explore this result, we show in Fig. 5e–f the power received and the BER as a function of the position of the obstacle, in the cases with no phase plate (grey), a steering phase plate (green) and a caustic phase plate (blue). For a range of obstacle locations, the caustic beam outperforms conventional beam steering in terms of received power (Fig. 5e) and BER (Fig. 5f). It should be noted that the same waveform was used in these comparative measurements, which means that it may not be compensating for any phase dispersion introduced using the caustics beam. A detailed analysis of the effects of chromatic dispersion is included in Supplementary Note 5. Compensation of chromatic dispersion by waveform tuning might result in an improvement of the quality of the received signal and bite-error rate. Finally, this experimental result is not specific to the particular semi-infinite metal obstacle shown in Fig. 5; we also observe improved performance using a caustic beam in the case of other types of obstacles (see Supplementary Note 6).

A familiar ray optics description, such as that used to schematically illustrate the definition of a caustic beam in Fig. 1, might suggest that the obstacle in these experiments is entirely blocking the radiation emitted by some portion of the transmitting aperture (the phase plate), and that the received signal originates only from the remaining (unobstructed) portion. If this was the case, then sliding a metal knife edge directly in front of the phase plate would have no effect on the received power until the knife edge reaches the unobstructed portion, which still has a line-of-sight view of the receiver. However, this simple view fails to capture the complexity of near-field behavior, which is not typically well described by ray optics. This is demonstrated in Fig. 5g, which shows the power drop (blue points) at the receiver as the transmitting aperture is progressively blocked. The vertical dashed line shows the location where the knife edge begins to block line-of-sight rays. The drop in received power to the left of this vertical line shows that even the portions of the aperture which have no line-of-sight view contribute to the signal at the receiver. Thus, although geometric optics can be used to engineer the trajectory of a caustic beam (as discussed above), a more careful treatment is required to understand the coupling of power from a transmitter to a receiver.

## Conclusion

The results presented above demonstrate the unique and valuable possibilities of operating wireless networks with near-field links. These results do not purport to show a solution to all blockage problems, however they clearly expand the range of possibilities for engineered wavefronts that can overcome obstacles without relying on non-line of sight paths. We show for the first time that it is possible to close a data link using a self-accelerating beam with a curved trajectory at THz frequencies, illustrating its benefits for obstacle avoidance. We provide the first analysis of the limitations of the bandwidth of such transmission imposed by curvature. This curvature dispersion leads to both frequency-dependent amplitude and phase responses at the receiver. Our discussion above focused on the effect of amplitude (Fig. 4) since that has a greater impact on our measurements. However, the frequency-dependent phase can also play a role in limiting data rates (see Supplementary Note 5), especially for wideband signals. We also experimentally show that a caustic beam is superior to a line-of-sight beam for obstacle avoidance. Finally, we provide the tools needed to assess near-field link budgets, and we show that self-accelerating beams outperform simple steered Gaussian beams in the near field.

These results show that trajectory engineering of near-field wave fronts will be an important tool in future physical layer implementations. However, it is clear that a great deal of additional research will be required to fully realize the benefits of near-field networking with self-accelerating beams. For example, we have emphasized the fact that different beams are optimal for establishing links in different conditions. This is true in cases where there is no obstacle in the beam path, but even more so when an obstacle moves through the direct line-of-sight path. Just like in cases of beam steering with phased arrays, sensing capabilities will be required in order for a transmitter to determine the precise parameters of the curved beam needed to close the link; this obviously depends on the obstacle size, type and shape. One can envision that base stations will incorporate the ability to hop from one wave front to the next, as situations change^52^. Obviously, the static amplitude and phase control apertures used for demonstration purposes in our study will need to be replaced by devices that can be dynamically reconfigured, of which several have been recently reported in the literature^45,51,53,54^. Lastly, curved *up-link* transmission (from the user to the base station) can yield additional challenges not considered here: while laptops and tablets have sufficiently large form factors to enable the apertures discussed herein, devices such as phones and VR/AR glasses have smaller and non-planar form factors that must be considered. Despite these considerations, our results clearly demonstrate that wireless networks operating above 100 GHz will derive substantial benefit from leveraging the power of near-field wave front engineering.

## Methods

### Experimental system

As mentioned, the aperture size is an important factor in realizing trajectories with tighter bending. To create this electrically large input aperture in our experiments, we use a Galilean beam expander design, in which a positive and negative lens increase the beam spot size. The beam is then incident on a phase plate or metasurface (described below) that generate the accelerating wave front in a transmission geometry. Two systems are used to measure the generated accelerating beams: a broadband THz time-domain spectrometer (TDS) and a narrow-band system based on a frequency multiplier chain. The TDS system is comprised of two fiber-coupled photoconductive antennas excited by fs-optical pulses, in which an optical delay line is used to retrieve the time-dependent THz electric field with a bandwidth spanning 0.1-3 THz. The receiver is mounted on a mechanical stage and scanned in two dimensions to obtain the spatially varying radiation profile in the near field (Fig. 2c) as a function of frequency (Fig. 4a).

The other source employed in our experiments consists of a frequency multiplier chain (multiplication factor of 16) driven by a 12.5 GHz signal, modulated using a double balanced mixer (limited bandwidth of 500 MHz). The system is driven by a pulse pattern generator outputting up to 1.2 Gbps on-off keying (OOK) signal with a pseudo-random binary sequence of length ${2}^{7}-1$. We note that OOK modulation is one of the preferred physical layer modes of the IEEE 802.15.3d standard^55^. The signal is then received by a horn antenna-coupled Schottky diode, and passes through low-pass filters, and can then be routed to a real-time bit-error rate tester (BERT), an oscilloscope (for eye diagrams) or a power meter. We verified through experiments that the caustic beam showed in Fig. 5 could transmit data rates up to 1.2 Gbps with BER <${10}^{-3}$. The heat map shown in Fig. 2a was obtained with this system by moving a metallic sheet in the beam path and measuring the power on a fixed detector located 1 meter away and 10 cm aside from the input aperture (knife-edge measurement). The heat map was then calculated by taking the derivative along $x$ of the measured results.

### Fabrication of the phase masks and metasurfaces

In this work, the accelerated beams were generated in transmission using two different methods: plastic phase plates and metasurfaces. Phase plates have the advantage of imposing a spatially continuous phase profile that can better replicate the calculated phase-only caustic profile. In contrast, metasurfaces produce a discretized version of the spatially-varying profile, but have the ability to also control the amplitude of the transmitted beam, to realize an Airy function for example.

The phase plates are 3D-printed using fused-deposition modeling in which the phase is locally varied by changing the thickness of the 3D-printed material, in our case polylactic acid (PLA). The incurred phase, after propagation through a thickness $h$, is $\phi \left(x\right)=2\pi \left(n-1\right)h(x)/\lambda$, with $n=1.6$ is the refractive index of PLA which is virtually frequency independent in the THz band (measured with a THz-TDS spectrometer). Using Eq. 1 for a caustic beam we can calculate the required phase profile, and the corresponding height profile of the phase plate. To realize a beam steered at an angle $\theta$, we use a phase of the form $\phi \left(x\right)=2\pi x\sin \theta /\lambda .$ The phase is wrapped over the $0-2\pi$ range to achieve a compact phase plate. We note that the propagation through the PLA material introduces very little absorption losses^56^. In our experiment, a $2\pi$ propagation at 200 GHz (1.3 mm thickness) introduces only ~1 dB losses.

The metasurfaces are fabricated with the hot stamping technique introduced in^57^. This technique allows the prototyping of metallic metasurfaces on a paper substrate. In brief, the design is printed using a standard laser printer, before being transferred to a sheet of paper using a laminator and commercially available aluminum-based foil. Importantly, this technique can be used to produce large area metasurfaces, limited in size by the typical letter size of a sheet of paper. As mentioned, this is important because large input apertures are required to increase the curvature of the bending beam. Our metasurface design uses a C-shape meta-element arranged in a square lattice, with a spacing of 0.72 mm, and a total size of 20 ×15 cm^58^. By varying the radius and opening of the C-shape across the metasurface, we can realize a cross-polarized arbitrary phase and amplitude profile in transmission. For example, we showed in^59^ that this fabrication technique was effective in fabricating large area beam steering metasurfaces.

### Supplementary information

Supplementary information

Description of Additional Supplementary Files

Supplementary Video 1

## Data Availability

All relevant data are available from the corresponding author upon reasonable request.
