# Supplementary material for: Curving THz wireless data links around obstacles
Source: Commun Eng. 2024 Mar 30;3:58. doi: 10.1038/s44172-024-00206-3 (PMC10981670; doi:10.1038/s44172-024-00206-3)
Supplement: Supplementary file 1 — Supplementary information [file 44172_2024_206_MOESM1_ESM.pdf]

Supplementary material for: Curving THz wireless data links around obstacles

Hichem Guerboukha[1], Bin Zhao[2], Zhaoji Fang[3], Edward Knightly[2], and Daniel M. Mittleman[3]
[1]School of Science and Engineering, University of Missouri-Kansas City, Kansas City MO, USA
[2]Department of Electrical and Computer Engineering, Rice University, Houston TX, USA
[3]School of Engineering, Brown University, Providence RI, USA

Supplementary Note 1. Blockage comparison of a Gaussian beam and a caustic beam.

In this Supplementary section, we show simulations building on the results from Fig. 1 of the main text. Here, we compare the performance of a caustic beam (Fig. S1a) to that of a Gaussian beam (Fig. S1b) and a focused beam (Fig. S1c) for the blockage situation illustrated in Fig. 1. We note that these finite element method simulations were performed using the same input power at the input aperture, which allows us to make a fair comparison between the different cases. Notice the difference in color scale, which indicates that the field is more localized on the aperture in the caustic case compared to the Gaussian and focused cases. Assuming an input power of 0 dBm, the caustic simulation reveals -1.4 dBm of power at the receiver, while the Gaussian simulation yields -7.3 dBm, and the focused beam shows -10 dBm. In all instances, the received power is determined by integrating the intensity across the receiver's aperture, which, in this case, is the cell phone held by the person. These simulations illustrate that using a caustic beam in this specific geometry enables the receiver to capture a lot more power from the transmitter compared to the other two scenarios.

A closer examination of the field distribution helps explain these findings. The caustic beam is more spatially concentrated at the receiver; only 1.4 dBm of power is lost elsewhere. This contrasts with the large Gaussian beam, which directs a substantial portion of its power (7.3 dBm) either towards the man's head or the floor. One might assume that simply focusing the beam on the receiver could increase the receiver power without the need for caustics beams. However, as evidenced when comparing the caustic to the focused beam, attempting to focus the beam on the receiver results in a great amount of power (10 dBm) being blocked by the man's head. In fact, in this situation, the focused beam gives even worse results than the Gaussian beam. This result highlights that caustic beams surpass conventional beam focusing, thanks to trajectory engineering, which enables to design paths that avoid otherwise blocking obstacles.

The important losses in beam focusing in this specific scenario can be understood geometrically: the ray connecting the center of the aperture to the center of the receiver passes through the man's head, resulting in the blockage of an important amount of the beam (~10 dB). One could compare an alternative situation where this is not the case anymore. In Fig. S2, we replicate the simulations shown in Fig. S1, but this time with an aperture half the size, positioned on the right side of the original aperture. In this new configuration, the steered Gaussian beam (Fig. S2b) exhibits improved performance (5.5 dB power loss), compared to the Gaussian beam that utilizes the full aperture (7.3 dB power loss, Fig. S1b). This improvement is the result of having less energy absorbed by the man; an important portion still reaches the floor and contribute to losses. In contrast, the caustic beam (Fig. S2a) performs even better than when using the full aperture, with only 0.64 dB of the power loss. Similarly, the focused beam (Fig. S2c) shows comparable results (only 0.84 dB losses) since this time the wave is not obstructed by the man's head. In summary, these results confirm two key findings: 1) In the absence of obstacles, the caustic beam performs as effectively as a focused beam. 2) However, in the presence of obstacles, the caustic beam outperforms the focused beam because its trajectory has been engineered to circumvent obstructions.

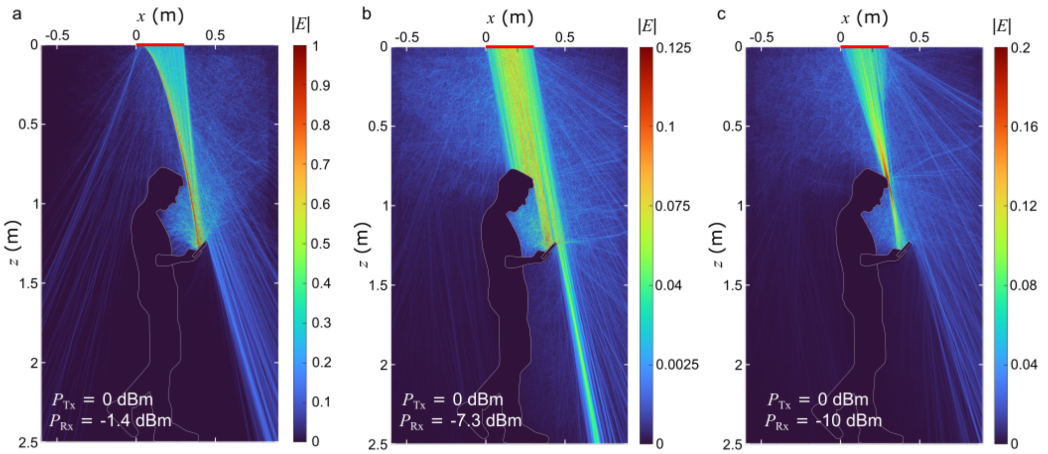

Fig. S1. Comparison of Gaussian, caustics and focused beam with a full aperture (a) Finite element method simulation for the caustic beam described in Fig. 2 for an aperture of 30 cm, (b) for a steered Gaussian beam, and (c) for a steered focused beam. All simulations consider the same input power at the input aperture. Note the difference in color scale of $|E|$ between (a), (b), and (c). For simplicity, the human silhouette is made of water ($\varepsilon = 5.39 + 5.98j$).

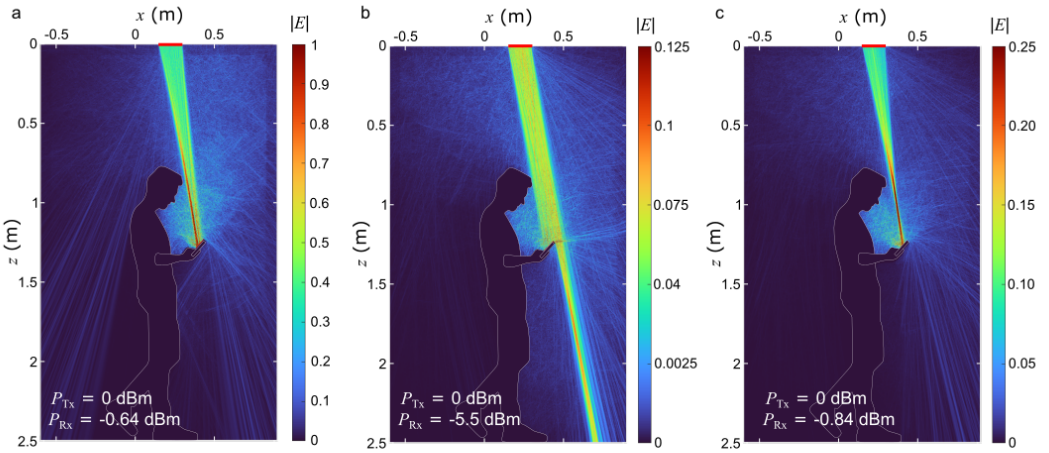

Fig. S2. Comparison of Gaussian, caustics and focused beam with a half aperture (a) Finite element method simulation for the caustic beam described in Fig. 2 for an aperture of 15 cm, (b) for a steered Gaussian beam, and (c) for a steered focused beam. All simulations consider the same input power at the input aperture. Note the difference in color scale of $|E|$ between (a), (b), and (c). For simplicity, the human silhouette is made of water ($\varepsilon = 5.39 + 5.98j$).

Supplementary Note 2. Required Aperture Size for a Given Trajectory

Let $g(z)$ be the function that describes the propagation in free space in the z direction (bold black curve in Fig. S3). We wish to generate the function between the two points $\big(z_1, g(z_1)\big)$ and $\big(z_2, g(z_2)\big)$, both located on the $g(z)$ curve, and indicated respectively by the red and dot circles in Fig. S3. The caustics are defined from the tangents with

$$\tan\theta = \frac{dg(z)}{dz} \tag{S1}$$

For example, consider a point $(z_i, g(z_i))$ of index i on the tangent (grey circle in Fig. S3). Its associated caustic can be mathematically written as the linear line:

$$c_i(z) = \left.\frac{dg(z)}{dz}\right|_{z=z_i} z + g(z_i) - \left.\frac{dg(z)}{dz}\right|_{z=z_i} z_i \tag{S2}$$

shown as a dotted grey line in Fig. S3. In particular, the terms

$$c_i(0) = g(z_i) - \left.\frac{dg(z)}{dz}\right|_{z=z_i} z_i \quad \text{(S3)}$$

correspond to the intersection of the caustic with the input plane $z = 0$. Given two points on the trajectory $(z_1, g(z_1))$ and $(z_2, g(z_2))$, we can calculate their caustics and find the distance between them on the input plane. This corresponds to the required size of the input aperture:

$$D_x = |c_2(0) - c_1(0)| = \left|\left(g(z_2) - \left.\frac{dg(z)}{dz}\right|_{z=z_2} z_2\right) - \left(g(z_1) - \left.\frac{dg(z)}{dz}\right|_{z=z_1} z_1\right)\right| \text{ on } t \quad \text{(S4)}$$

Eq. S4 is a simple formula that can be used to extract the required input size to realize a particular trajectory. For example, applying this to the parabolic trajectory realized in Fig. 2a ($g(z) = -0.0001z^2 + 0.12z + 9.82$, where $g(z)$ and z are in mm) between the experimentally realized ranges of $z_1 = 0$ m and $z_2 = 0.9$ m, we require $D_x \sim 45$ mm, which is what we used in the experiment. If we wish to maintain the parabolic shape on a larger range, say $z_2 = 2$ m, then we would require $D_x = 226$ mm.

The graphical illustration shown in Fig. S3 indicates limitations in realizing trajectories with sharp bending. For these trajectories, the caustic intersects the input axis ($z = 0$) at large values of x, implying the need to have a large input aperture. This imposes a constraint on the practicality of generating such beams.

Finally, as mentioned in the main paper, the restriction on the caustic beam is that the trajectory must be convex. This means that the caustics intersect the trajectory only once, at the tangent, and at no other points along the curve. Fig. S3 is an example of convex trajectory, while its inset shows a counterexample of a non-convex trajectory (polynomial of order 3). Polynomials of odd orders have zigzag shapes, and therefore do not comply with the convex requirement. However, it is still possible to construct S-shaped beams by subdividing the aperture in multiple parts such that each part contributes to one convex portion of the curved shape[1].

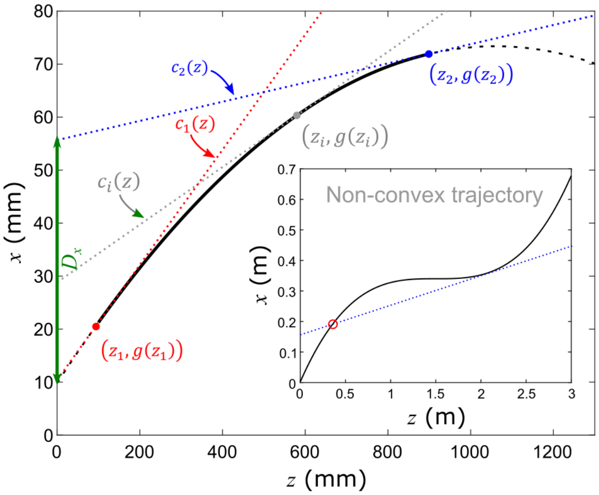

Fig. S3. Aperture size in relation to the desired trajectory. Inset shows an example of a non-convex trajectory of expression $x = 0.1(z - 1.5)^3 + 0.34$ (z in meters) that cannot be natively realized with caustics. An illustrative caustic shown as the dotted blue line intersect the curve at a second point (indicated by the red circle).

Supplementary Note 3. Near Field Link Budget Calculation

In this section, we show how to calculate link budgets in the near field to reproduce the results shown in Fig. 3a. We assume a cartesian coordinate system where the input aperture is

located on the $z = 0$ plane. A coordinate on the input aperture is denoted $(x_1, y_1, 0)$, while a coordinate on the observation aperture is (x_2, y_2, z_2). Assuming a field profile at the input plane $E_1(x_1, y_1)$, the profile at the observation plane can be found using the Huygens-Fresnel diffraction integral:

$$E_2(x_2, y_2, z_2) = \frac{1}{j\lambda z_2} \iint_{S_1} E_1(x_1, y_1, 0) \frac{\exp(jk\,|\vec{r}_{12}|)}{|\vec{r}_{12}|^2} dx_1 dy_1 \quad \text{(S5)}$$

where the integral is performed over the input aperture, denoted S_1. Following the Huygens-Fresnel principle, this integral corresponds to a summation of infinitesimal spherical wavelets $\exp(jkr)/r$. The distance between an input point and an observation point is given by:

$$|\vec{r}_{12}| = \sqrt{(x_2 - x_1)^2 + (y_2 - y_1)^2 + z_2^2} \quad \text{(S6)}$$

This scalar approximation is valid when assuming a linear, isotropic, homogeneous and non-dispersive medium (e.g., air)[2]. We also assume that we are in the radiative near field i.e., wavelengths away from the antenna to avoid any reactive near field effects such as surface waves, back-coupling into the antenna, or coupling between the different vector components of the electromagnetic field.

In practice, we note that Eq. S5 requires extensive computational power, especially for large aperture sizes compared to the wavelength as expected in near-field THz channels. For example, a 10-cm square aperture at 300 GHz, a 1-cm square observation aperture, and a $\lambda/2$ discretization of the integral would require summation of >16 million points.

To compute the integral faster, we use the Fresnel approximation which allows to write the $|\vec{r}_{12}|$ term as

- In the denominator: $|\vec{r}_{12}| \approx z$
- In the exponential: $|\vec{r}_{12}| \approx z\left[1 + \frac{1}{2}\left(\frac{x_2 - x_1}{z}\right)^2 + \frac{1}{2}\left(\frac{y_2 - y_1}{z}\right)^2\right]$ (S7)

With this, Eq. S5 becomes:

$$E_2(x_2, y_2, z_2) = \frac{e^{jkz_2} e^{\frac{jk}{2z}(x_2^2 + y_2^2)}}{j\lambda z} \iint_{S_1} E_1(x_1, y_1, 0) \exp\left(\frac{jk}{2z}(x_1^2 + y_1^2)\right) \exp\left(-\frac{jk}{z}(x_1 x_2 + y_1 y_2)\right) dx_1 dy_1 \quad \text{(S8)}$$

which can then be separated in two integrals:

$$E_2(x_2, y_2, z_2) = I_x(x_2, z) I_y(y_2, z) \quad \text{(S9)}$$

where

$$I_x(x_2, z_2) = \frac{e^{\frac{jkz_2}{2}} e^{\frac{jk}{2z}x_2^2}}{\sqrt{j\lambda z}} \int_{-D_x/2}^{D_x/2} E_1^x(x_1, z = 0) \exp\left(\frac{jk}{2z_2} x_1^2\right) \exp\left(-\frac{jk}{z_2} x_2 x_1\right) dx_1 \quad \text{(S10)}$$

$$I_y(y_2, z_2) = \frac{e^{\frac{jkz_2}{2}} e^{\frac{jk}{2z}y_2^2}}{\sqrt{j\lambda z}} \int_{-D_y/2}^{D_y/2} E_1^y(y_1, z = 0) \exp\left(\frac{jk}{2z_2} y_1^2\right) \exp\left(-\frac{jk}{z_2} y_2 y_1\right) dy_1 \quad \text{(S11)}$$

and where for symmetry, we distributed the square root of the multiplicative term in front of the integral. Using these symmetries, we can solve $I_x(x_2, z_2)$ independently from $I_y(y_2, z_2)$, and construct the 3D field using Eq. S9. In our implementation, $I_x(x_2, z_2)$ is solved numerically from the input aperture E_1^x e.g., phase profile to generate a given caustic beam or a complex amplitude Airy profile. As for $I_y(y_2, z_2)$, for the link budget results shown in Fig. 3a, we assume a Gaussian beam with a full width at half maximum of 50 mm (in power) as E_1^y. From the calculated radiated field E_2, one can measure the power that traverses an aperture S as:

$$P = \frac{1}{2Z_0} \int_S |E_2(x, y)|^2 \, dS \quad \text{(S12)}$$

where $Z_0 = \sqrt{\mu_0/\varepsilon_0} \sim 377\ \Omega$ is the free space impedance with ε_0 and μ_0 respectively the vacuum permittivity and permeability. We can therefore obtain the Friis ratio as:

$$\frac{P_{\mathrm{Rx}}}{P_{\mathrm{Tx}}} = \frac{\iint |E_2(x_2, y_2, z_2)|^2 dS_{\mathrm{Rx}}}{\iint |E_1(x_1, y_1, 0)|^2 dS_{\mathrm{Tx}}} \quad \text{(S13)}$$

In practice, the integral over the transmitter is taken over a surface that is very close to the transmitter and very large, such that all the energy emitted by the transmitter aperture is accounted for.

In general, the received power depends on the coupling between the transmitted field and the radiated field of the receiver (when operated as a transmitter by the reciprocity principle). The power coupling between the two fields is generally expressed as the efficiency term, a number between 0 and 1, indicating respectively no coupling and total coupling of the energy. For the calculations shown in Fig. 3a, we assume perfect coupling (efficiency of 1). Therefore, the power obtained from Eq. S12 is the maximum power that a receiver could obtain. In other words, we assume that the receiver is capable of perfect synthetic aperture and realize any phase/amplitude profile that matches the incoming field (no impedance mismatch). While this assumption may be unrealistic, it allows our analysis to remain agnostic with respect to the type of receiver that is used.

Supplementary Note 4. A Note on the Definitions of Near-field and Far-field Regions

As mentioned in the main paper, curved beams exist in the near-field region of the transmitter antenna aperture. In this section, we provide additional discussion regarding this statement. First, it is interesting to note that there are different definitions of the near-field regime, depending on the electromagnetic community. In particular, we consider the rf definition (predominantly used at lower frequencies) and the optics definition (used at high optical frequencies). It is important for us to highlight the distinctions between the two, because the THz band is spectrally located in between, in a region often referred to as quasi-optics.

The conventional definition used by the rf community stipulates that the far-field begins when a spherical wave emitted from a point source located at a distance z results in a planar field on the receiver aperture of size D. This occurs when the phase difference between the aperture center and its edge is smaller than an arbitrarily chosen value of $\pi/8$. It can be demonstrated that this condition holds for distances $z > 2D^2/\lambda$. Therefore, receivers located beyond this distance are in the far-field, while those located nearer are in the near-field. This definition is particularly useful when considering coupling efficiency of the radiated field to the receiving antenna, as a plane wave is generally desirable for efficient coupling. It is important to note that the antenna definition relies on assumptions about the *receiver's* characteristics; here, D corresponds to the receiver's aperture size. It also explicitly relies on an assumption that the radiated field is a spherical wave, which of course need not be true in the near field of an arbitrary source.

In contrast, under the definition generally used in optics, the far field is defined on the basis of the *transmitter* aperture. To derive the far-field distance, one begins with the Huygens-Fresnel diffraction integral (Eq. S5). After applying the Fresnel approximation (Eq. S8), one can observe that the integral closely resembles the two-dimensional spatial Fourier transform of the input aperture. To make the Fourier transform exact, an additional approximation is used. Referred to as the Fraunhofer approximation, this approximation aims to eliminate the quadratic terms in the complex exponential $\exp(jk(x_1^2 + y_1^2)/2z)$. This occurs when $z \gg k(x_1^2 + y_1^2)_{\max}/2$, in which case $\exp(jk(x_1^2 + y_1^2)/2z) \to 1$. Then, Eq. S8 becomes the exact Fourier transform (up to a multiplicative term):

$$E_2(x_2, y_2, z_2) = \frac{e^{jkz_2} e^{\frac{jk}{2z}(x_2^2+y_2^2)}}{j\lambda z} \iint_{S_1} E_1(x_1, y_1, 0) \exp\left(-\frac{jk}{z}(x_1 x_2 + y_1 y_2)\right) dx_1 dy_1 \qquad \text{(S14)}$$

As mentioned, the Fraunhofer approximation occurs when the distance to the aperture is $z \gg k(x_1^2 + y_1^2)_{\max}/2$. There, the term $(x_1^2 + y_1^2)_{\max}$ corresponds to the square of the transmitter aperture radius, or $(D/2)^2$. At distances greater than $z > \pi D^2/2\lambda$, the radiated field is the Fourier transform of the input field.

The antenna and optics definition differ only by the value of the constant in front of the D^2/λ term, which are, of course, very close to one another: factor of 2 for the antenna, and factor of $\pi/2 \approx 1.57$ for the optics definition. This is not surprising, since the two discussions are describing essentially the same physics. More important than the value of the constant is that these two definitions stem from fundamentally different perspectives: the rf definition is

based on the wavefront at the receiver, while the optics definition derives from the wavefront transmitted by the aperture.

In the context of this paper, we focus on the transmitter, and therefore used a near-field optics approach. It is crucial to point out that in the optical near-field, waves need not be planar, nor spherical. Wave fronts can be very complicated, and this complexity can be leveraged for near-field wavefront engineering. To illustrate this complexity, consider the electric field of an Airy beam in the near-field region, as simulated in Fig. S4. At a fixed distance from the aperture, the field oscillates between positive (red) and negative (blue) values in a non-spherical and non-planar fashion. Indeed, multiple lobes can be observed, and two adjacent lobes have conjugate phases ($+\pi$ and $-\pi$). The definition of the near field employed by the optics community is more appropriate for discussing such waves as it does not rely on any assumption about the shape of the wave front.

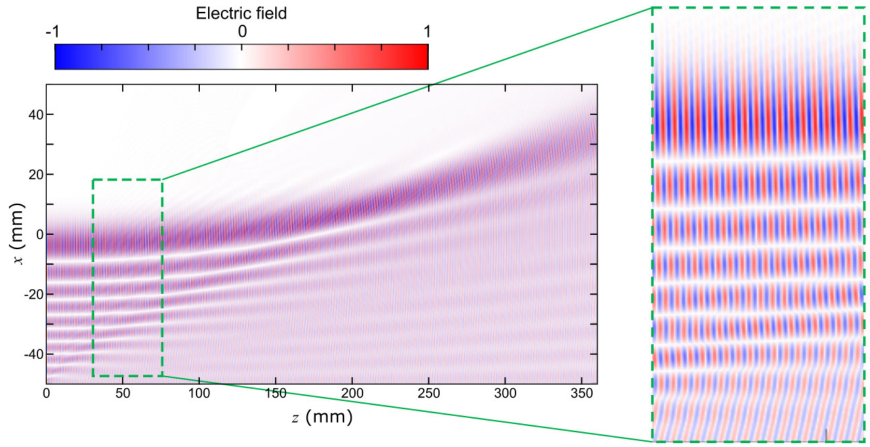

Fig. S4. Airy beam electric field distribution in the near-field region of a transmitter. In this full-wave simulation, the frequency is 180 GHz, and the transmitter aperture is 60 mm.

Supplementary Note 5. Theoretical Bandwidth of an Airy beam

As discussed in[3], the field distribution of a 1D finite-energy Airy beam is:

$$E(x,z) = \mathrm{Ai}\left(\frac{x}{x_0} - \frac{z^2}{4k_0^2 x_0^4} + i\frac{az}{k_0 x_0}\right)\exp\left[i\frac{z}{2k_0 x_0^2}\left(\frac{x}{x_0} - \frac{z^2}{6k_0^2 x_0^4} + a^2 x_0^2\right)\right]\exp\left[a(x - \frac{z^2}{2k_0^2 x_0^3})\right] \quad \text{(S15)}$$

where $\mathrm{Ai}(x)$ is the Airy function defined as:

$$\mathrm{Ai}(x) = \frac{1}{\pi}\int_0^\infty \cos\left(\frac{t^3}{3} + xt\right) dt \quad \text{(S16)}$$

Here, z is the propagation axis, and x the transverse coordinate. The truncation factor a ensures containment of the field and finite energy, while x_0 is a transverse scale that controls the propagation trajectory of the Airy beam. Eq. S15 is a solution to the paraxial wave equation; as such, an Airy beam can be generated by imposing a phase and profile at the input plane $z = 0$:

$$E(x,0) = \mathrm{Ai}\left(\frac{x}{x_0}\right)\exp\left(\frac{ax}{x_0}\right) \quad \text{(S17)}$$

We now derive the bandwidth as a function of the propagation for a receiver located on the trajectory and operating at a frequency f_0. We note that when the truncation parameter is small ($a \to 0$), the propagated field is diffraction-less, and follows the trajectory defined by setting the argument of the Airy function to 0:

$$x(z) = \frac{z^2}{4k_0^2 x_0^3} \quad \text{(S18)}$$

In this case ($a \to 0$), the full width at half maximum of the main lobe of the Airy beam does not change as it propagates. We can calculate its value numerically. First, we note that the first maximum of an Airy function corresponds to the first zero of its derivative, which is located at $x = -1.01879$. Then, the peak value (in power) is $[\text{Ai}(-1.01879)]^2 = 0.2870$. The full width at half maximum can therefore be obtained by finding the zeros of the following equation:

$$[\text{Ai}(x)]^2 - \frac{0.2870}{2} = 0 \tag{S19}$$

This equation contains many solutions, and we are interested only by the first two, which correspond to the main lobe. They are $x_1 = -0.092$ and $x_2 = -1.722$. This means that the full width at half maximum is $D = |x_1 - x_2| = 1.630$.

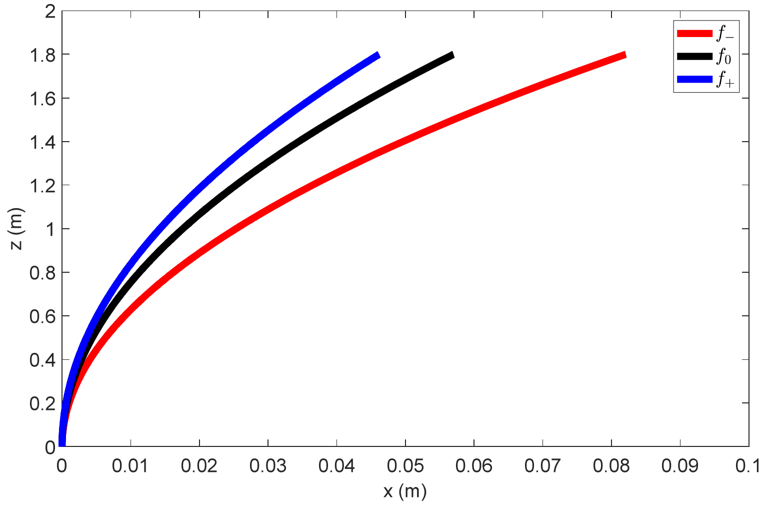

Fig. S5. Bandwidth construction using trajectories. Trajectories of three illustrative frequencies of curvature $x_0 = 0.01$: f_-, f_0 and f_+ are respectively 150, 180 and 200 GHz.

With this value of the full width at half maximum, finding the bandwidth along the trajectory is a geometric problem. Consider an Airy beam of frequency f_0 defined by the trajectory $x(z) = z^2/4k_0^2x_0^3$ (black line in Fig. S5). We are interested in finding the bandwidth along this trajectory. For this, we look for the two adjacent frequencies f_- and f_+ that generate trajectories below and above the f_0 trajectory (red and blue curves in Fig. S5 respectively). These frequencies have the edge of their beamwidth spatially positioned on the trajectory f_0, and can be mathematically found by solving

$$\frac{z^2}{4k_-^2x_0^3} - \frac{D}{2} = \frac{z^2}{4k_0^2x_0^3} \tag{S20}$$

for the frequency below ($k_- = 2\pi f_-/c$), and

$$\frac{z^2}{4k_+^2x_0^3} + \frac{D}{2} = \frac{z^2}{4k_0^2x_0^3} \tag{S21}$$

for the frequency above ($k_+ = 2\pi f_+/c$). There, $D = 1.63x_0$ is the beamwidth as shown above.

From this, we can calculate the bandwidth as

$$\Delta f(z, x_0) = |f_+ - f_-| = \frac{zf_0}{\sqrt{z^2 - 2k_0^2x_0^3D}} - \frac{zf_0}{\sqrt{z^2 + 2k_0^2x_0^3D}} \tag{S22}$$

We can also invert this equation and write the range z of a given bandwidth:

$$z(\Delta f, x_0) = \left(\frac{4D^2k_0^4}{\Delta f^2(4f_0^2 - \Delta f^2)}\left(2f_0^4 - \Delta f^4 + 2f_0^2\Delta f^2 + 2f_0^4\sqrt{\frac{2\Delta f^2}{f_0^2} + 1}\right)\right)^{1/4} x_0^{3/2} \tag{S23}$$

It is also interesting to explore the phase response at the receiver to characterize any potential chromatic dispersion. While an exhaustive study of the phase response exceeds the scope of this work, we present some preliminary results in the following. For this purpose, we fix the frequency at 180 GHz and set the curvature parameter $x_0 = 0.01$ m (black curve in Fig. S5). Considering a point receiver positioned along the trajectory at $x = 0.0508$ m, $z = 1.7$ m, we calculate its phase using the Airy beam formula in Eq. S15. The phase over the main bandwidth is shown in Fig. S6b and reveals a clear nonlinear behavior indicative of chromatic dispersion. We fit the phase to a second order polynomial centered around $\omega_0 = 180$ GHz:

$$\phi(\omega) = p_2(\omega - \omega_0)^2 + p_1(\omega - \omega_0) + p_0 \quad \text{(S24)}$$

The obtained fitting parameters are $p_0 = 1.584$, $p_1 = 11.73$ ps, and $p_2 = -20.32$ ps^2. In particular, the second order fitting coefficient p_2 is recognized as the group delay dispersion (GDD), which can lead to temporal broadening of the pulse and consequent inter-symbol interference in communications[4,5].

Based on the results of Fig. S6, we can estimate the impact of this temporal dispersion on the maximal achievable bitrate[6,7]. For that purpose, we first compute the group velocity dispersion (GVD) as the second order derivative of the propagation constant:

$$\text{GVD} = \frac{d^2\beta}{d\omega^2} = \frac{1}{L}\frac{d^2\phi}{d\omega} \quad \text{(S25)}$$

where L is the distance between the source and the considered point in the near field. Here, we assume $L = 1.7$ m. Fig. S6c shows the computed GVD for the geometry and the bandwidth considered in Fig. S6a. Expressed in units of $\text{ps} \cdot \text{THz}^{-1} \cdot \text{cm}^{-1}$, GVD corresponds to ps of time broadening at a given THz frequency and for a given propagation length in cm. From this, we can calculate the maximal bitrate for an amplitude shift keying (ASK) modulation format (on-off keying, OOK) as[8]:

$$B_{\max} = \frac{1}{4\sqrt{|\text{GVD}|L}} \quad \text{(S26)}$$

Based on these results, for an operation frequency of 200 GHz, the maximal bitrate for an OOK modulation format (when GVD is the only limiting factor) is ~45 Gbps. We emphasize that this is an example of calculation for the specific Airy beam mentioned here, and that these results might change for other configurations. Furthermore, strategies for compensating chromatic dispersion exist[9,10]; we defer their exploration to future work.

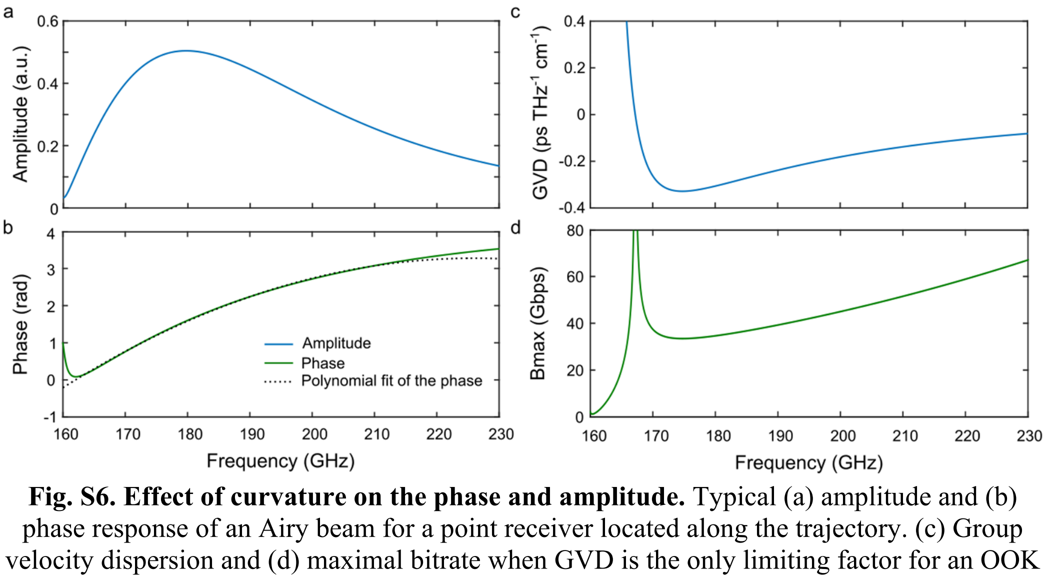

Fig. S6. Effect of curvature on the phase and amplitude. Typical (a) amplitude and (b) phase response of an Airy beam for a point receiver located along the trajectory. (c) Group velocity dispersion and (d) maximal bitrate when GVD is the only limiting factor for an OOK modulation format. In this example, we are considering an Airy beam with a parameter $x_0 =$ 1 cm, and a point receiver located at $x = 0.0508$ m, $z = 1.7$ m.

Supplementary Note 6. Additional measurements showing obstacle evasion of Airy beams
In this section, we show additional experimental and simulation measurements showing how self-accelerating beams can evade obstacles in the beam path. In the following, we assume a 12-mm diameter cylindrical object made of water ($\varepsilon = 5.39 + 5.98j$) with its axis perpendicular to the xz propagation plane. To reproduce the obstacle experimentally, we use a straw filled with water. We compare a steered Gaussian beam to the Airy beam used in Fig. 2b. These are generated using two different metasurfaces fabricated with the hot-stamping method explained in the Methods section. Fig. S7a and Fig. S7b show the simulated fields in the absence of obstacle for the steered Gaussian and Airy beam respectively. The red dot indicates the position of the receiver (350 mm away from the metasurface, and along the parabolic trajectory).

The obstacle is placed at $z = 120$ mm and moved along the x axis. Fig. S7c shows the power measured by a point receiver (blue star) as a function of the x-position of the obstacle, both experimentally and with simulations. For the Gaussian beam, the obstacle creates two dips in the measured power. The power increase between the two dips is caused by edge diffraction. In comparison, the Airy beam has an asymmetric extinction profile related to the asymmetry of the radiated near field. When the obstacle is between x values of -38 mm and -20 mm, the Airy beam outperforms the Gaussian. At $x = -30$ mm, the performance is maximal. The fields displayed in Fig. S7d (Gaussian) and Fig. S7e (Airy) show this situation, illustrating how Airy beams can recover through self-healing, a property that allows the beam to reconstruct itself downstream from obstacles.

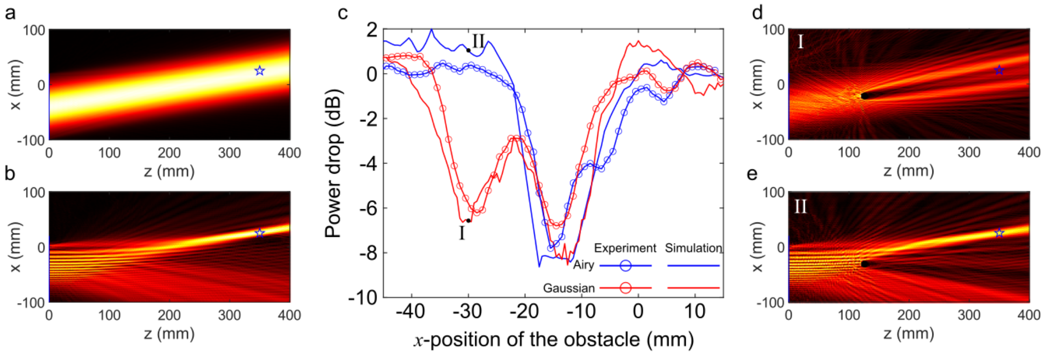

Fig. S7. Evasion of small obstacles with Airy beam (a) Simulated intensity of a steered Gaussian beam and (b) an Airy beam. The blue star indicates the position of the receiver. (c) Experiment (circles) and simulation (line) comparison of the power at the position of the receiver when the obstacle is moved along the x-axis at $z = 120$ mm. (d) Simulated intensity when the obstacle is at $x = -30$ mm for the steered Gaussian beam and (e) for the Airy beam.

Supplementary References

1. Zhu, L., Wang, A. & Wang, J. Free-space data-carrying bendable light communications. *Sci. Rep.* **9**, (2019).
2. Goodman, J. W. *Introduction to Fourier Optics, Second Edition*. (McGraw-Hill, 1996). doi:10.1117/1.601121.
3. Efremidis, N. K., Chen, Z., Segev, M. & Christodoulides, D. N. Airy beams and accelerating waves: an overview of recent advances. *Optica* **6**, 686 (2019).
4. Strecker, K., Ekin, S. & O'Hara, J. F. Fundamental Performance Limits on Terahertz Wireless Links Imposed by Group Velocity Dispersion. *IEEE Trans. Terahertz Sci. Technol.* **12**, 87–97 (2022).
5. Fang, Z. *et al.* Secure Communication Channels Using Atmosphere-limited Line-of-sight Terahertz Links. *IEEE Trans. Terahertz Sci. Technol.* (2022) doi:10.1109/TTHZ.2022.3178870.
6. Guerboukha, H., Shrestha, R., Neronha, J., Fang, Z. & Mittleman, D. M. Conformal leaky-wave antennas for wireless terahertz communications. *Commun. Eng.* **2**, 17

(2023).
7. Nallappan, K. *et al.* Terahertz Waveguides for Next Generation Communication Network. in *Next Generation Wireless Terahertz Communication Networks* (ed. S. Ghafoor, M. H. Rehmani, and A. D.) 379–410 (CRC Press, Taylor and Francis Group, 2021). doi:10.1201/9781003001140-18.
8. Agrawal, G. P. Signal propagation in fibers. in *Lightwave technology: telecommunication systems* 63–106 (Wiley, 2005).
9. Ma, T., Nallapan, K., Guerboukha, H. & Skorobogatiy, M. Analog signal processing in the terahertz communication links using waveguide Bragg gratings: example of dispersion compensation. *Opt. Express* **25**, 11009 (2017).
10. Strecker, K., Ekin, S. & O'Hara, J. F. Correction of channel dispersion in terahertz wireless communications. 22 (2022) doi:10.1117/12.2559120.
