# Supplementary material for: Curving THz wireless data links around obstacles
Source: Commun Eng. 2024 Mar 30;3:58. doi: 10.1038/s44172-024-00206-3 (PMC10981670; doi:10.1038/s44172-024-00206-3)
Supplement: Supplementary file 2 — Description of Additional Supplementary Files [file 44172_2024_206_MOESM2_ESM.pdf]

# Description of Additional Supplementary Files

**File name:** Supplementary Video 1

**Description:** Real-time experiment showing the effectiveness of curved beams to communicate around an obstacle. The receiver is initially located in the non-line-of-sight, and no signal is received. Then, a phase plate with a linear phase profile is introduced in the beam which directs the beam towards receiver, producing a clear eye diagram. Next, a metallic plate is moved into the beam, partially blocking it, and distorting the eye diagram. Finally, the linear phase plate is replaced with a phase plate generating a caustic beam designed to curve around the metallic plate.
